# Finerenone: Potential Clinical Application Across the Spectrum of Cardiovascular Disease and Chronic Kidney Disease

**DOI:** 10.3390/jcm14093213

**Published:** 2025-05-06

**Authors:** Nowreen Haq, Pulkita Uppal, Taslova Abedin, Anuradha Lala

**Affiliations:** 1 University of Maryland Affiliated Practice, Baltimore, MD 21201, USA; 2 Luminis Health Arundel Medical Center, Annapolis, MD 21401, USA; 3 Anne Arundel Medical Center, Annapolis, MD 21401, USA; 4 University of Maryland School of Medicine, Baltimore, MD 21201, USA; ttasinar@gmail.com; 5 Mount Sinai, Fuster Heart Hospital, Icahn School of Medicine, New York, NY 10029, USA; anu.lala@mountsinai.org

**Keywords:** cardiovascular disease, chronic kidney disease, finerenone, hyperkalemia, mineralocorticoid receptor antagonist, type 2 diabetes

## Abstract

Type 2 diabetes (T2D) is the leading cause of chronic kidney disease (CKD) and is a risk factor for progression to end-stage kidney disease and cardiovascular morbidity and mortality. Despite pharmacologic treatment, residual risk of disease progression and adverse outcomes remains substantial. Finerenone is a nonsteroidal mineralocorticoid receptor antagonist (MRA) approved in the United States for use in patients with CKD associated with T2D. The present review focuses on finerenone use, including its pharmacologic basis, indication and eligibility, and practical aspects of incorporation into routine clinical practice (particularly primary care). Results from the two placebo-controlled phase 3 clinical trials of finerenone (plus maximum tolerated dose of a renin-angiotensin-aldosterone system inhibitor) in patients with CKD associated with T2D showed a significantly lower risk of CKD progression and cardiovascular events with finerenone versus placebo. These effects of finerenone were applicable across the broad spectrum of patient participants, including those with baseline comorbidities such as a history of heart failure or a history of atherosclerotic cardiovascular disease. We also compare finerenone to steroidal MRAs and discuss the relevance of ongoing and recently completed clinical trials of finerenone in other patient groups, which could expand finerenone use further to a broader spectrum of patients.

## 1. Introduction

Type 2 diabetes (T2D) is a leading cause of chronic kidney disease (CKD) [1] and a risk factor for progression to end-stage kidney disease (ESKD) [2]. Persistently high blood glucose levels (hyperglycemia) in T2D can cause kidney and cardiovascular (CV) injury due to the effects of interrelated hemodynamic dysregulation, metabolic disturbances, and inflammation, which are key drivers for the development and progression of CKD in T2D [3,4,5,6,7]. Furthermore, due to the interconnectivity between the kidneys, metabolic processes, and the heart in normal state and in disease [8], both CKD and T2D are linked with CV morbidity and mortality [9,10,11]. Risk for CKD progression and subsequent complications is generally captured and tracked by measurements of kidney function via the estimated glomerular filtration rate (eGFR) and the extent of kidney damage measured by the degree of albuminuria [12,13]. By virtue of these assessments, patients who may benefit from specific treatments are identified to improve outcomes [12,13,14]. Management of CKD associated with T2D generally involves both nonpharmacologic (lifestyle optimization) and pharmacologic approaches to optimize glucose and blood pressure control, as well as preventing or reducing proteinuria in order to reduce the risks of CKD progression and CV events [12,15]. However, despite these management approaches, there is a continuing residual risk for the development and progression of CKD associated with T2D [16], which may further translate into poor quality of life and increased risk of hospitalization and death.

Finerenone is a nonsteroidal mineralocorticoid receptor antagonist (ns-MRA) that can be used to treat patients who have CKD associated with T2D [17]. Results from two large placebo-controlled phase 3 clinical trials (total randomized = 13,171) showed that when used in combination with a renin–angiotensin–aldosterone system (RAAS) inhibitor, finerenone reduced the risk of sustained loss of kidney function, ESKD, and CV events in adult patients with CKD associated with T2D [18,19]. Finerenone is indicated in the US for the treatment of CKD associated with T2D [17].

In this review, we discuss finerenone in the context of these phase 3 clinical trials in CKD associated with T2D, which included a broad spectrum of patient participants. We also look at practical considerations for incorporating finerenone into routine clinical practice, such as serum potassium monitoring for hyperkalemia and combination therapies with finerenone, depending on patient need. Because finerenone is different from steroidal MRAs, we compare finerenone to this drug class and also discuss RAAS inhibitors, which were used in combination with finerenone and placebo in the phase 3 clinical trials. We believe that focusing on these specific aspects of finerenone will be particularly helpful to those who work in primary or general care medicine. Finally, we consider finerenone’s potential application in other patient populations where clinical trials have recently been completed or are still ongoing, which may extend the spectrum of eligible patients further. [This article includes a plain language summary included as a Supplementary File S1].

## 2. Methods

PubMed and non-PubMed searches were used to find applicable articles for inclusion in the review. Three separate PubMed searches were completed, and all search outputs were limited to article Title only, English, and the past 10 years. Limitations by article type were applied differently across the three searches. Search 1. (“Finerenone” OR “Kerendia” OR “BAY94 * OR BAY 94 *); excluded letters, editorials, and case studies. Search 2. (“Spironolactone”) OR (“Eplerenone”); limited to phase 2 and phase 3 trials, randomized controlled trials, and meta-analyses/systematic reviews. Search 3. (“Mineralocorticoid receptor antagonist” OR “MRA”); limited to review articles and meta-analyses/systematic reviews. The search results were downloaded from PubMed into the EndNote 9.3.3 program, where the articles were screened using EndNote’s search features as follows: removal of duplicate articles; review of article titles and removal of nonrelevant articles; review of full text and removal of nonrelevant articles. Further articles were added to the total number if recommended by the authors and if new data or important analyses had been published since the original searches had been completed.

Non-PubMed searches included direct access of clinical practice guidelines from associated websites, such as www.kdigo.org/guidelines/ (accessed on 6 January 2025) and www.diabetesjournals.org/care (accessed on 6 January 2025). Drug product labels were accessed from the FDA website (www.fda.gov/drugs (accessed on 6 January 2025). Articles may have also been removed after the original searches if they were deemed no longer relevant. The method used for searching clinicaltrials.gov is provided in Section 11.

## 3. CKD Associated with T2D Represents a Broad Spectrum of Patients

Diabetes is common, and its prevalence is expected to increase. Globally, it was estimated that there were 529 million people living with diabetes in 2021, corresponding to 6.1% of the world’s population at the time; T2D cases made up 96% of all diabetes cases, with an age-standardized prevalence of 5.9% in 2021 [20]. T2D prevalence is projected to rise by 61.2%, and it is estimated the condition will affect over 1.2 billion people in 2050. In the US, around 37 million people have diabetes, which comprises 11.3% of the US population, with some people living with undiagnosed diabetes [21]. T2D is a leading cause of CKD and CV disease worldwide, with approximately 40% of people with T2D developing CKD [1]. Around one-third of people with CKD also have a CV disease comorbidity [10]. Of CV disease comorbidities, heart failure (HF) with preserved ejection fraction (HFpEF) and HF with reduced ejection fraction (HFrEF) occur in ~17% and ~7% of patients with T2D, respectively [22]. Thus, given the interlinking of the heart and vasculature with the kidneys in both normal state and pathologic state, it is unsurprising that CKD associated with T2D is associated with a broad spectrum of patients.

## 4. Defining CKD

The development of CKD and/or CV disease as a complication of T2D is mostly caused by the presence of hyperglycemia over a prolonged period, which leads to a chronic inflammatory state in the kidneys and associated vasculature [3,4,6,23,24,25]. CKD is diagnosed if there is a persistent (>3-month) elevation of urinary albumin excretion (albuminuria), low eGFR, or other manifestations of kidney damage [13,15]. Alongside a diagnosis, CKD is staged based on cause, eGFR category, and albuminuria category (measured via urine albumin-to-creatinine ratio [UACR]) [13]. There are six CKD stages: stages 1, 2, 3a, 3b, 4, and 5 [12,13]. Stage 1 is the least severe (damage and loss of kidney function is minimal), whereas stage 5 is the most severe and represents ESKD, often necessitating a kidney transplant or long-term dialysis. However, it is important to note that many people with stage 1 or 2 CKD may not know they have CKD because the condition can be asymptomatic [26], which emphasizes the need for routine screening of all people with T2D [12,15]. According to a consensus report from Kidney Disease: Improving Global Outcomes (KDIGO) and the American Diabetes Association, patients eligible for treatment of CKD associated with T2D are those with at least moderately increased albuminuria (UACR ≥ 30 mg/g) [12]. This includes patients categorized as stage 1 or 2 based on their eGFR [12]. Guidelines indicate the importance of treating patients at risk for CKD progression. Therefore, the presence of albuminuria helps inform treatment decisions in addition to eGFR as per KDIGO risk categories, as an increase in UACR and decrease in eGFR together is associated with a 20-fold increased risk of progression to ESKD compared with no changes in either measure [27].

**Figure 1:**
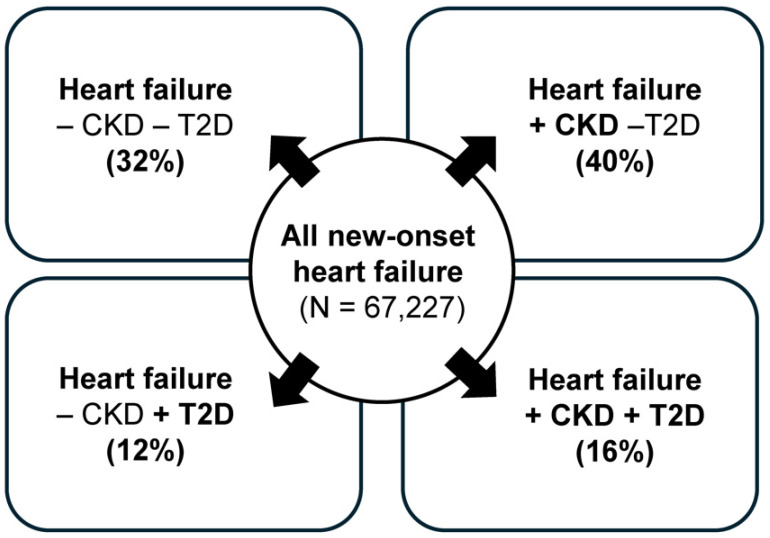
Interconnection between heart failure (HF), chronic kidney disease (CKD), and type 2 diabetes (T2D). Figure based on results from Lawson et al. [28]. ‘New onset’ refers to the first year following a HF diagnosis. Ejection fraction data for HF were not collected, and HF stage data were not collected. CKD was diagnosed based only on the estimated glomerular filtration rate value (urine albumin-to-creatinine ratio data were not collected).

The damage caused by chronic hyperglycemia to the kidneys and CV system in T2D, CKD, and CV disease (such as HF) is interlinked; this means that having one of these conditions increases the risk of developing the other (Figure 1) [28], or the worsening of one may worsen the other. Although current treatments have substantially reduced the speed of CKD and/or CV disease progression, as well as risk, vs. what was previously available, it is important to note that most treatments still have a residual risk for disease progression, which varies according to the drug or drug combination used [18,19,29,30,31,32]. It is also important to note that having significant kidney dysfunction itself precludes many therapies that may otherwise help improve kidney function. Another consideration is that T2D and CKD represent a broad patient population in terms of severity, as well as additional comorbidities and risk factors. These points highlight the need for additional and improved treatment strategies or novel treatments in this high-risk population.

## 5. Finerenone Is a ns-MRA: Differences vs. Steroidal MRAs

Overactivation of the mineralocorticoid receptor (MR) has an important role in the pathogenesis of kidney and CV diseases. Signaling via the MR is essential for electrolyte and fluid homeostasis in the kidneys, although other functions are suggested by its presence in other cells throughout the body [33]. Under pathologic conditions, overactivation of the MR “switches” it from a homeostatic regulator to a pathophysiologic mediator by promoting oxidative stress, inflammation, and fibrosis [34]. Progressive glomerular and tubular injury within the kidneys through continued disrupted metabolic pathways and the influx of proinflammatory and profibrotic mediators result in albuminuria and progressive reduction in GFR and eventual ESKD [35,36,37]. The rationale for MR blockade is that it should stop these pathologic pathways and slow the progression of kidney and CV disease. In preclinical animal models of CV and kidney disease, finerenone has been shown to prevent inflammation and fibrosis (often mediated by oxidative stress) and subsequent disease progression [38,39,40].

**Figure 2:**
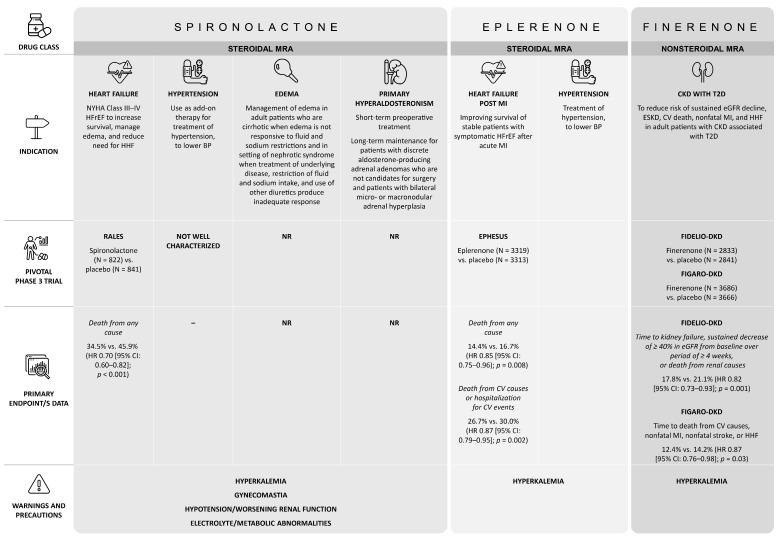
Indication and pivotal trials overview for MRAs. BP: blood pressure; CKD: chronic kidney disease; CV: cardiovascular; eGFR: estimated glomerular filtration rate; ESKD: end-stage kidney disease; HFrEF: heart failure with reduced ejection fraction; HHF: hospitalization for heart failure; HR: hazard ratio; MI: myocardial infarction; MRA: mineralocorticoid receptor antagonist; NYHA: New York Heart Association; NR: not reported; T2D: type 2 diabetes.

There are steroidal MRAs (spironolactone, eplerenone) and ns-MRAs—the latter of which includes finerenone (currently the only ns-MRA approved for use in the US) (Figure 2) [17,18,19,41,42,43,44]. MRAs can help mitigate tissue inflammation and end-organ damage by inhibiting MR overactivation [45,46]. However, steroidal MRAs and ns-MRAs have notably different properties and clinical indications. One such difference is that the nonsteroidal structure/unique binding of finerenone strongly inhibits cofactor recruitment to the MR and modifies proinflammatory and profibrotic gene pathways independently of aldosterone, unlike steroidal MRAs [39,46,47].

Spironolactone and eplerenone differ from finerenone in molecular structure, pharmacokinetics, and pharmacodynamics [33,46,47]. Spironolactone is a nonspecific MRA and also has an affinity for progesterone, androgen, and glucocorticoid receptors. Blockade of these other receptors with spironolactone reflects the increase in sex-related side effects such as gynecomastia, sexual dysfunction, and abnormal menstruation with this drug [33,41]. Spironolactone and eplerenone both increase cortisol levels and hemoglobin A_1c_ (HbA_1c_), although the metabolic effects differ between the two drugs [48]. Eplerenone is chemically different from spironolactone and, consequently, has greater selectivity for MRs and minimal binding to progesterone, androgen, and glucocorticoid receptors; therefore, the frequency of sexual side effects is low [42]. Finerenone also has high selectivity and high potency for the MR; it is at least as potent as spironolactone but more potent than eplerenone and is more selective than both steroidal MRAs [33,46,47]. MRA therapy can cause hyperkalemia. All three MRAs include a warning in their labels for potential risk of hyperkalemia [17,43,44]. People with reduced kidney function and risk factors for higher potassium levels are at greater risk of developing hyperkalemia when taking MRAs, although hyperkalemia may be managed for many patients using serum potassium monitoring and dose adjustments. Clinical practice guidelines also provide guidance on which patients to select for treatment with finerenone (patients with a normal serum potassium concentration) and to monitor serum potassium regularly after initiation of treatment [14]. Greater tissue distribution in the kidneys, as observed with the two steroidal MRAs, may suggest a greater potential for hyperkalemia than for finerenone, where there is a more balanced distribution across myocardial and renal tissue. This explains why finerenone also demonstrates protective effects in both organs [33,38,45,46].

Based on phase 3 data, spironolactone is approved in the US for HFrEF as an add-on therapy for hypertension, for the treatment of primary hyperaldosteronism, and for the management of edema (Figure 2) [43]. Eplerenone is also indicated for HFrEF, but after an acute myocardial infarction (MI) and for hypertension, for which it may be used alone or in combination with antihypertensive agents (Figure 2) [44]. However, neither of these steroidal MRAs is approved for use in patients with CKD and T2D, as there are no supporting phase 3 outcomes data. In phase 2 trials, they reduced albuminuria in various populations, including those with CKD associated with T2D [49,50,51,52,53,54]. In both the finerenone phase 3 trials, finerenone or placebo was administered in combination with a RAAS inhibitor. In the next section, we discuss the importance of RAAS in blood pressure control, how RAAS inhibitors are treatments for hypertension, and their role in CKD treatment.

## 6. The RAAS, Its Inhibition, and the Finerenone Phase 3 Clinical Trials

Patients included in the phase 3 clinical trials were receiving finerenone (or placebo) in combination with the maximum tolerated dose (MTD) of a RAAS inhibitor. The RAAS has an important role in the homeostasis of blood pressure and blood volume in the body [55]. An active component of the RAAS pathway is the hormone angiotensin II, which is produced in response to low blood pressure/blood volume [55,56]. Some of the most significant effects of angiotensin II are that it causes the reabsorption of sodium (and consequently also the reabsorption of water by osmosis) in the kidneys, as well as causing vasoconstriction, which has the collective effect of increasing blood volume and blood pressure [56]. Angiotensin-converting enzyme inhibitors (ACEis) and angiotensin receptor blockers (ARBs) are types of RAAS inhibitors and are used clinically to reduce blood pressure and albuminuria [14,15,57]. Both these drug types inhibit the production of, or action of, angiotensin II [58,59].

In CKD, pathologic narrowing of the efferent arteriole caused by dysregulated release of angiotensin II and vasodilation of the afferent arteriole caused by vasodilatory stimulators such as high blood sugar levels, increase the pressure inside the glomerulus, resulting in intraglomerular hyperfiltration [60]. Over time, such hyperfiltration causes many downstream pathologic effects, including enhanced excretion of large proteins into urine, such as the protein albumin (called albuminuria) [13,61]. RAAS inhibitors reduce albuminuria by causing vasodilation of the efferent arteriole via inhibitory action on angiotensin II [15,60,62]. RAAS inhibitors have been part of the standard of care in CKD (where there is albuminuria) for many years. Indeed, RAAS inhibitors are recommended by KDIGO (2022) as the first-line treatment of albuminuria with and without hypertension in people with diabetes (at the MTD). In the finerenone phase 3 clinical trials, a RAAS inhibitor was included alongside a placebo or finerenone due to the proven cardiorenal benefits of RAAS inhibitors in people with albuminuria, T2D, and/or elevated blood pressure.

## 7. Potential Benefits of Finerenone Across the Spectrum of Patients with T2D and CKD

Two large phase 3 placebo-controlled clinical trials tested the efficacy and safety of finerenone in combination with the MTD of a RAAS inhibitor in patients with CKD associated with T2D. Patients were required to have T2D as defined by the American Diabetes Standards of Care in Diabetes (2010 version) and a diagnosis of CKD based on prespecified criteria [18,19]. A total of 13,171 patients were randomized, and 12,990 (98.6%) completed the trials. Patients assigned to the placebo arm took a RAAS inhibitor at the MTD plus placebo; patients assigned to the finerenone arm received finerenone at either 10 or 20 mg/day (starting dose depending on eGFR; target dose was 20 mg/day) plus a RAAS inhibitor at the MTD. Serum potassium level had to be ≤4.8 mmol/L at the screening visit to be eligible and was monitored throughout both trials.

One of the two trials (FIDELIO-DKD: Finerenone in Reducing Kidney Failure and Disease Progression in Diabetic Kidney Disease) focused on the kidney effects of finerenone (primary composite outcome: kidney failure or death from kidney causes) [18]. CV effects of finerenone were a secondary outcome (secondary composite outcome: death from CV causes, nonfatal MI, nonfatal stroke, or hospitalization for HF [HHF]) [18]. After a median follow-up of 2.6 years following finerenone or placebo treatment, finerenone resulted in a lower risk of CKD progression risk and CV events than placebo. Table 1 provides details of the primary and secondary outcome results and adverse events of special interest from the FIDELIO-DKD trial [18,19,63,64,65,66,67,68,69,70,71,72]. The other trial (FIGARO-DKD: Finerenone in Reducing Cardiovascular Mortality and Morbidity in Diabetic Kidney Disease) focused on CV effects of finerenone (primary composite outcome: death from CV causes, nonfatal MI, nonfatal stroke, or HHF) [19]. Kidney effects of finerenone were a secondary outcome (first secondary composite outcome: kidney failure or death from kidney causes) [19]. After a median follow-up of 3.4 years following finerenone or placebo treatment, finerenone improved CV outcomes compared with placebo. Table 1 provides details of the primary and secondary outcome results and adverse events of special interest from the FIGARO-DKD trial.

### 7.1. Patient Pretreatment Clinical Characteristics and Patient Subgroups in the Finerenone Phase 3 Clinical Trials

Patient participants were required to have CKD and T2D within prespecified bounds depending on which trial they were participating in; other patient baseline (pretreatment) characteristics included a mean duration of diabetes of 15.4 years, and a mean HbA_1c_ of 7.7% across both trials poor blood glucose control (HbA_1c_ > 12%) was an exclusion criterion [63]. Additionally, the majority of patients (>97%) were taking a glucose-lowering therapy, with insulin the most commonly taken (58.6%), followed by a glucagon-like peptide-1 receptor agonist (GLP-1 RA) (7.2%) and/or a sodium-glucose co-transporter-2 (SGLT2) inhibitor (6.7%). In addition to a RAAS inhibitor, over 50% of patient participants were also taking a diuretic. Many patients also had American College of Cardiology/American Heart Association stage 1 hypertension (mean systolic blood pressure [SBP] 136.7 mmHg); uncontrolled hypertension was an exclusion criterion [57,63]. Approximately 45% had a history of CV disease [18,19], and patients with chronic symptomatic HF were excluded from both trials [63].

Subanalyses (or secondary analyses) of data from phase 3 clinical trials focus on patient subgroups so that the effects of a drug or drugs according to disease-specific characteristics can be evaluated. Several subanalyses of data from the FIDELIO-DKD and FIGARO-DKD clinical trials have been conducted. Subanalyses using data from the FIDELIO-DKD clinical trial include evaluation of finerenone efficacy and safety according to baseline glucose control (HbA_1c_ < 7.5% vs. ≥ 7.5%; insulin use, yes vs. no) [64]; with or without a history of HF [65]; with or without a history of atrial fibrillation (AF) or flutter (AFF) [66]; and by baseline office SBP quartiles [67]. The main concluding point from these subanalyses was that finerenone improved cardiorenal outcomes irrespective of baseline HbA_1c_ level or insulin use [64], HF history at baseline [65], AFF history at baseline [66], and baseline office SBP [67]. Subanalyses using data from the FIGARO-DKD clinical trial included evaluating finerenone’s efficacy and safety in patients with or without a history of HF [68] and finerenone effect by UACR subgroups (moderately increased and severely increased UACR at baseline) [69]. The main concluding point from these two subanalyses from the FIGARO-DKD clinical trial was that finerenone improved cardiorenal outcomes regardless of baseline HF history [68] and that finerenone may protect against CKD progression and CV events in patients with T2D and early or late-stage CKD [69]. More details on the results of these patient subgroups in the FIDELIO-DKD and FIGARO-DKD clinical trials are provided in Table 1, and the Section 8 will further explore the results from the analyses involving the subgroups of patients with and without history of CV disease at baseline.

### 7.2. Pooling of Patient Data from the Finerenone Phase 3 Clinical Trials

A prespecified pooled analysis of the data from both phase 3 trials was conducted (the FIDELITY analysis), and several secondary analyses of these data were conducted (Table 1). The results of the FIDELITY pooled analysis provide further support for the potential kidney and CV benefits of finerenone in patients with CKD associated with T2D. This prespecified pooled analysis was conducted to provide more robust estimates of finerenone efficacy and safety across the spectrum of patients with CKD associated with T2D and to provide a degree of precision to the findings that were not possible to obtain by considering each trial separately [63]. Kidney outcomes assessed in the FIDELITY pooled analysis (N = 13,026) were a primary kidney composite of (1) time to first onset of kidney failure (defined as ESKD [initiation of chronic dialysis for ≥90 days or kidney transplantation] or sustained decrease in eGFR to <15 mL/min/1.73 m^2^), (2) sustained ≥ 57% decrease in eGFR from baseline over ≥4 weeks, or (3) renal death. A secondary composite kidney outcome was also assessed in the pooled analysis and comprised (1) time to first occurrence of kidney failure, (2) sustained ≥40% decrease in eGFR from baseline over ≥4 weeks, (3) renal death, (4) time to all-cause mortality, (5) time to all-cause hospitalization, and (6) change in UACR from baseline to month 4. A sustained decrease in eGFR (≥57%, equivalent to doubling of serum creatinine) was selected in the FIDELITY analysis. It has historically been used as an outcome in diabetic nephropathy studies and is a more robust kidney failure surrogate outcome than a ≥40% decrease (which was used in phase 3 clinical trials), particularly when initial changes in eGFR occur. For both kidney composite outcomes, risk was significantly reduced by 23% (≥57% eGFR decrease composite) and 15% (≥40% eGFR decrease composite) with finerenone treatment compared with placebo (Table 1).

As well as potential kidney protective effects, the effect of finerenone on CV outcomes was also assessed in the FIDELITY pooled analysis, with the main CV outcome defined as (1) time to death from CV causes, (2) nonfatal MI, (3) nonfatal stroke, or (4) HHF [63]. For this composite endpoint, risk was significantly reduced by 14% with finerenone treatment compared with placebo (Table 1). A deeper analysis of the CV outcomes of the FIDELITY population, specifically looking into causes of mortality, demonstrated that finerenone significantly reduced the risk of all-cause and CV mortality vs. placebo, with these findings consistent across KDIGO risk categories (low, moderate, high, and very high risk of CKD progression) [12,70]. As part of this analysis, two populations were analyzed: the intention-to-treat (ITT) population—which is standard in randomized, controlled trials and estimates the effect of drugs in all randomized patients (regardless of treatment adherence). Additionally, an on-treatment analysis was used, which included events that occurred while on treatment and for ≥30 days after the last intake of the study medication, to estimate the effect of the drugs while being taken by patients [70]. Risk for all-cause mortality and CV mortality was significantly reduced with finerenone treatment in the on-treatment analysis, whereas risk in the ITT analysis (the overall population) was not significantly different between finerenone and placebo. This may highlight a need for good adherence to optimize the benefit of finerenone in clinical practice. Incidence of death from renal causes was low in this population (<0.1% in both groups), and there was no significant difference between finerenone and placebo for non-CV/nonrenal mortality (Table 1). Other secondary analyses of the primary data have been conducted to assess the benefit of finerenone across CKD stages and KDIGO risk categories (Table 1).

### 7.3. CKD Risk Stages at Baseline in the Finerenone Phase 3 Clinical Trials

Patients eligible for finerenone treatment of CKD associated with T2D are distributed across the KDIGO risk categories CKD stages 1–4 [14,15,73]. Guideline recommendations and the findings from the finerenone phase 3 trials in patients with CKD associated with T2D taken together suggest that a broad spectrum of patients may be eligible for treatment with finerenone. This distribution of KDIGO risk categories was illustrated in the FIGARO-DKD phase 3 clinical trial, which revealed that most patients (82%) were at high risk of CKD progression, 17% at moderate risk, and nearly 1% at low risk, based on eGFR and normal to mildly increased albuminuria [19]. Both phase 3 trials [18,19] assessed similar populations of patients who had a diagnosis of CKD associated with T2D. Both trials also included patients with similar definitions of CKD, split into two severity groups. In the FIDELIO-DKD clinical trial, patients had persistent, moderately elevated albuminuria (UACR 30 to <300 mg/g) and an eGFR of 25 to <60 mL/min/1.73 m^2^ or persistent, severely elevated albuminuria (UACR 300–5000 mg/g) and an eGFR of 25 to <75 mL/min/1.73 m^2^ (mean baseline eGFR was 44.3 mL/min/1.73 m^2^ and median baseline UACR was 852 mg/g) [18]. There was a slight variation in the CKD definitions in the FIGARO-DKD trial, whereby patients were eligible with an eGFR of 25 to 90 mL/min/1.73 m^2^, with UACR 30 to <300 mg/g or an eGFR of ≥60 mL/min/1.73 m^2^ with UACR 300 to 5000 mg/g (mean baseline eGFR was 67.8 mL/min/1.73 m^2^ and median baseline UACR was 308 mg/g) [19]. The disparity between the studies shows that those taking part in the FIDELIO-DKD trial had CKD stages 2 to 4, whereas those taking part in the FIGARO-DKD had CKD stages 1 to 4 (based on eGFR criteria). This highlights the broad spectrum of patients who entered these two pivotal studies. In a subanalysis of the FIGARO-DKD data, the benefit of finerenone on the risk of CKD progression favored those with severely increased albuminuria (UACR ≥ 300 mg/g) compared with those with moderately increased albuminuria (UACR 30 to <300 mg/g). However, an interaction was only observed in the kidney composite that included the sustained decrease in eGFR ≥ 40% [69]. These findings suggest that patients with more advanced diseases may derive a greater benefit from finerenone. However, similar analyses of CV outcomes stratified by moderately or severely increased albuminuria show that the effect of finerenone was consistent with the overall population, indicating that finerenone may benefit a wider selection of patients [69,71]. Kidney protective outcome results from the pooled FIDELITY analysis were further supported in a focused analysis [74]. In this analysis, treatment with finerenone demonstrated a consistent kidney protective effect across the spectrum of CKD severities, including those with worse baseline kidney function (eGFR < 45 mL/min/1.73 m^2^ and UACR ≥300 mg/g). Results from the two phase 3 finerenone clinical trials and clinical practice guideline recommendations both tell us which patients should be considered for finerenone treatment: those with various stages of kidney dysfunction and those with a wide range of albuminuria levels.

## 8. Cardiovascular Effects of Finerenone in CKD Associated with T2D

### 8.1. Systolic Blood Pressure

Patients with CKD associated with T2D are at high risk for CV disease [10]. Hypertension is a risk factor for developing both CKD and CV disease [75,76], supporting the need for regular blood pressure monitoring and management in high-risk individuals. Around two-fifths of patients with CKD associated with T2D also have hypertension (defined as SBP ≥ 140 mmHg), so it is important from a clinician’s perspective to see if there is any difference in effect based on the history of hypertension [77]. In a subgroup analysis of the FIDELIO-DKD clinical trial, with data stratified by SBP quartiles (≤128.7, >128.7 to ≤138.3, >138.3 to ≤148.0, >148.0 mmHg), finerenone had a small (statistically insignificant vs. placebo) effect on office SBP. However, finerenone’s cardiorenal benefits were consistent irrespective of baseline office SBP and were unaffected by arterial hypertension (Table 1) [67]. The authors of the analysis suggested that in addition to the reduction in BP, other mechanisms are likely to improve cardiorenal outcomes with finerenone [67].

### 8.2. Atherosclerotic CV Disease

CKD, along with T2D and hypertension, places patients at high risk for atherosclerotic CV disease (ASCVD) [78]. In an analysis of the FIDELITY pooled analysis, the efficacy and safety of finerenone in patients with or without ASCVD at baseline was evaluated [72]. ASCVD history (yes/no) was determined by prespecified medical loglines of carotid endarterectomy, coronary artery disease, MI, ischaemic stroke, and peripheral arterial occlusive disease. Close to half (45.6%) of patients included in the analysis had a history of ASCVD at baseline. These individuals were more likely to have a longer duration of T2D and a history of AF or coronary heart disease but were less likely to have hypertension vs. those without a history of ASCVD. The incidence of the composite CV outcomes of CV death or HHF and all-cause mortality was higher in patients with ASCVD history vs. those without; however, there was no difference between groups in the composite kidney outcome. In comparing finerenone with placebo in patients with and without ASCVD at baseline, finerenone reduced the risk of CV events and improved kidney outcomes consistently across the broad spectrum of patients with CKD and T2D, irrespective of prevalent baseline ASCVD (Table 1) [72].

### 8.3. Risk of Heart Failure

Approximately 15% of patients with CKD associated with T2D are living with HF [10]. Furthermore, when CKD and T2D are both present, a person’s risk of developing HF increases further due to the detrimental effects of worsening kidney function on the CV system [79]. Therefore, prevention of HF and other CV-related comorbidities in people with CKD and T2D is an important objective of treatment. In Table 1, we showed that finerenone was associated with a significant reduction in the risk of the composite endpoint of time to death from CV causes, nonfatal MI, nonfatal stroke, or HHF (*p* < 0.05 vs. placebo; both phase 3 clinical trials). Subanalyses of these data have shown that finerenone also reduces the risk of new-onset HF and remains effective in patients who reported a history of HF at baseline. In one secondary analysis of data from the FIGARO-DKD clinical trial, finerenone treatment was associated with significantly reduced (vs. placebo) HF-associated time-to-event outcomes (Table 1). This included a 29% reduction in the risk of first HHF and an 18% reduction in the composite of time to CV death or first HHF vs. placebo. This effect was independent of the history of HF. Furthermore, the risk of new-onset HF was 32% lower with finerenone compared with placebo [68]. Additionally, results from a subanalysis of the FIDELIO-DKD clinical trial showed that a history of HF at baseline in patients with CKD and T2D did not modify the response to finerenone in terms of CV and kidney outcomes [65]. This demonstrates the potential benefit of finerenone across a broad spectrum of patients, even those with more advanced diseases. Moreover, finerenone’s CV effect is relatively fast and may be sustained. Results from a subgroup analysis of the FIDELITY pooled analysis showed that HF-related outcomes (risk of first HHF, CV death or first HHF, recurrent HHF and CV death or recurrent HHF) might be reduced within weeks of initiating finerenone, with this benefit maintained for at least 4 years [71].

### 8.4. Atrial Fibrillation

People with CKD and T2D have an increased risk of developing AF [80]. The cause of AF in patients with CKD and T2D may be due to atrial structural or electrical remodeling that provides a precursor for the development of AF [81,82,83]. In an analysis of the FIDELIO-DKD clinical trial, the efficacy and safety of finerenone in patients with and without AFF at baseline were evaluated [66]. A total of 461 (8.1%) of patients involved in the FIDELIO-DKD trial had AFF at baseline. These individuals were more likely to be older, have a history of CV disease, and have a lower median UACR vs. those without a history of AFF. The risk of new-onset AFF was reduced by 29% in patients who received finerenone compared with those in the placebo group (hazard ratio [HR] 0.71 [95% CI: 0.53–0.94]; *p* = 0.0164) [66]. This analysis also demonstrated a consistent cardiorenal benefit with finerenone, irrespective of the history of AF.

## 9. Finerenone Use in Clinical Practice: Primary Care

Patients eligible for treatment with finerenone are those with CKD and T2D who are at risk of kidney disease progression and CV disease, assuming that there are no contraindications to treatment [14,15,17]. Primary care is an important point of access to finerenone for many patients with CKD associated with T2D. Primary care and general care providers are at the forefront of recognizing the symptoms and diagnosing CKD and have an important role in the management of CKD. However, given that early-stage CKD is often asymptomatic, patients may experience delayed diagnosis and have advanced disease at diagnosis, which is more complex and challenging to manage [14,84,85,86,87]. This makes routine (once per year) screening for CKD in people with T2D important and highlights the need for access to nephrology services, which is advocated by clinical practice guidelines [14,15,73,88].

For some patients, management of CKD might be suboptimal for reasons such as cost, limited or no access to a nephrologist or nephrology services, and clinical inertia [89,90,91]. Globally, rates of kidney disease and the provision of its care vary, with the most socially disadvantaged groups experiencing the greatest burden, although care for CKD in higher-income countries could also be improved [89,92]. In higher-income countries, governments tend to provide funding for only about half of the necessary CKD care. Although access to a nephrologist or nephrology services is better in higher-income countries, it lags when compared with access to other healthcare services. This may lead to patients not receiving appropriate care and treatment.

Prescribing patterns have revealed that cardiorenoprotective treatments may be underutilized in patients with T2D [93,94,95,96]. Data available on current prescribing patterns relate to the use of GLP-1 RAs and SGLT2 inhibitors, with limited published data available on prescribing patterns for finerenone. However, use of finerenone and where it may provide multisystem benefits should be further explored.

## 10. Finerenone Use in Clinical Practice: Serum Potassium Monitoring and Combination Therapy

As previously stated, finerenone is indicated in the US to reduce the risk of sustained eGFR decline, ESKD, CV death, nonfatal MI, and HHF in adult patients with CKD associated with T2D [17]. This section focuses on serum potassium monitoring because hyperkalemia (the clinical presentation of increased serum potassium) is an adverse event of special interest that can occur during finerenone treatment. An overview of adverse event data related to serum potassium levels in the finerenone phase 3 clinical trials is provided in Table 1. The recommended starting dose for finerenone is based on kidney function via eGFR and serum potassium levels. For patients with acceptable serum potassium levels, treatment with finerenone may be initiated at 10 mg once daily if eGFR is ≥25 to <60 mL/min/1.73 m^2^ and at 20 mg once daily if eGFR is ≥60 mL/min/1.73 m^2^; the target daily dose is 20 mg.

Finerenone should only be started in patients with serum potassium levels no greater than 5.0 mEq/L, given that hyperkalemia is a possible side effect of finerenone therapy [17]. Strategies to manage the risk of hyperkalemia include structured monitoring, temporary treatment interruption, and dose adjustment [97,98]. The prescribing information for finerenone recommends that eGFR and serum potassium are measured prior to initiation of treatment and that potassium levels are monitored during treatment [17]. Several risk factors for hyperkalemia have been identified for patients receiving finerenone. The greatest risk is a potassium level > 5 mmol/L and an eGFR < 25 mL/min/1.73 m^2^, although adding an SGLT2 inhibitor to finerenone may lower the risk for hyperkalemia [14,73,98]. Additional ongoing studies are investigating the effect of adding an SGLT2 inhibitor to finerenone plus RAAS inhibitor on predefined kidney outcomes (CONFIDENCE [NCT05254002], FLAMINGO [NCT05640180]; Table 2). These studies should provide further insights into the potential benefits of combining an SGLT2 inhibitor with an ns-MRA plus RAAS inhibitor in the management of CKD associated with T2D.

Guidelines advocate the use of combination therapy to maximize benefits for patients with CKD associated with T2D [14]. Finerenone should be taken alongside the MTD of a RAAS inhibitor, with or instead of an SGLT2 inhibitor (depending on patient need), although clinical practice guidelines state that more data are needed on combining MRA with other effective classes of medications, including SGLT2 inhibitors and GLP-1 RAs.

## 11. Unexplored Patient Populations and Areas for Future Research

A search of the database clinicaltrials.gov in April 2025 using the term “finerenone” with no other terms filtered resulted in 52 clinical studies being identified. After discarding completed trials for finerenone at any phase of development, the remaining 28 studies were hand-sorted for relevance to this section of ongoing finerenone studies.

We have already noted that in kidney and CV diseases, the MR pathway becomes overactivated through multiple mechanisms, and this represents a potential therapeutic target in CV disease [14]. Therefore, a rationale is presented for the investigation of finerenone in patients with CV disease, including HF. Finerenone is also being investigated in other cardiorenal indications in phase 3 clinical trials, and a study of HF (FINEARTS-HF [NCT04435626]) was completed in July 2024 (Table 2). Finerenone is also being tested in primary aldosteronism; four clinical studies are ongoing in this disease area (NCT05814770; NCT06457074; NCT06381323; NCT06164379). Additionally, finerenone is being tested in IgA nephropathy (NCT06580288), cardiovascular autonomic neuropathy (NCT06906081) and in patients who have received a radical nephrectomy for renal cell carcinoma (alone or in combination with empagliflozin) (NCT06818305).

Two observational studies (FINE-REAL [NCT05348733] and FIRST-2.5 [NCT06608212]) to assess real-life clinical practice and treatment patterns for patients with CKD associated with T2D who are prescribed finerenone are also underway. Both studies will collect demographic data for patients along with data on clinical characteristics and adverse events and information relating to other prescribed medications. In the FIRST-2.5 study, investigators will collect data on kidney function and possible heart problems to further assess the effectiveness of finerenone. The FIRST-2 (observational) trial (NCT05703880) was completed in 2024. Another observational study, which was recently completed, aimed to observe the demographics and clinical characteristics (comedications and comorbidities) of patients who have started or will start treatment for CKD or T2D (FINEGUST [NCT05526157]). FINEROD (NCT06278207) is an ongoing observational study taking place in Japan reviewing the safety and efficacy of finerenone in patients with CKD associated with T2D; data will be collected from commercial electronic health records and national claims data in Japan. IN-REALITY (NCT06763146) is an ongoing retrospective cohort study taking place in India reviewing the efficacy and safety of finerenone in people of Indian ethnicity diagnosed with CKD associated with T2D. Another real-world study is being conducted in patients with HFrEF without T2DM and CKD, as the effectiveness of finerenone in this population (beyond clinical phase 2) is currently unknown (NCT05974566). The hope is that these observational study data and others will provide real-world evidence to support the phase 3 clinical data.

## 12. Discussion

We discussed finerenone in our article in the context of completed phase 3 clinical trials and applicable analyses from these trials. For the trials involving patients with CKD associated with T2D (FIDELIO-DKD and FIGARO-DKD) this amounts to 13,171 randomized patients [18,19]. Finerenone is indicated for use in CKD associated with T2D. However, as with all medical decisions, it is important that clinicians consider factors such as a patient’s preexisting comorbidities and medications, possible contraindications, risk of side effects, and patient preference before deciding on drug treatments/s. We also acknowledge the importance of diverse evidence types for testing clinical efficacy and safety profiles. For finerenone, the current long-term safety data is relatively short for a chronic long-term condition (~2.6–3.4 years in the phase 3 trials), emphasizing the need for longer-term follow-up durations to assess the sustained effects and safety of finerenone over time. A limitation of the FIDELIO-DKD and FIGARO-DKD trials, and, therefore, also our data review, is that only 4.7% and 3.5% of participants, respectively, identified themselves as Black, thus limiting the generalizability of these data by race/ethnicity. We acknowledge the importance of real-world/observational studies in understanding the broader and longer-term effects of a drug, which is why we have included reference to completed and ongoing finerenone observational/real-world studies in our article. Results from these studies will add to existing data and help address some knowledge gaps.

Other drugs that can be used for the treatment of CKD associated with T2D and are included in the recent edition of the American Diabetes Association’s Standards of Care in Diabetes guidelines (with finerenone as a ns-MRA) include select SGLT2 inhibitors and the GLP-1 RA semaglutide [99]. Again, as noted above, treatment decisions should consider patient-related factors such as possible side effects, comorbidity, and contraindications. To further advance the diagnosis and treatment of patients with CV and kidney disease, novel biomarkers in the setting of CKD are needed to help improve outcomes for patients [100].

## 13. Conclusions

Finerenone is indicated for the treatment of patients with CKD associated with T2D, with treatment guidelines reflecting this, as well as providing a framework for how to manage this condition based on risk across a broad spectrum of patients. Within this framework, clinicians are guided on whom to treat, when to treat, when to refer for nephrology services, and how to treat patients. This review provided additional detail on these aspects with a focus on finerenone, which is an ns-MRA that has demonstrated significantly reduced kidney disease progression and reduced CV event risk across CKD stages in phase 3 trials. The treatment effect of finerenone was broadly unaffected by baseline CV comorbidities, demonstrating a cardiorenoprotective effect in patients with CKD and T2D, irrespective of hypertension or history of HF, AF, or ASCVD. It is important to note these aspects of finerenone efficacy were tested as sub- or secondary analyses from the two phase 3 clinical trials or the FIDELITY pooled analysis. Therefore, they do have limitations due to their smaller sizes. FIDELIO-DKD and FIGARO-DKD clinical trial enrollment criteria were applied to the US population to assess the characteristics of the eligible population and determine the number of individuals eligible for finerenone [101]. Potentially, 2.2 million individuals in the US could benefit from finerenone. The two finerenone phase 3 clinical trials are broadly generalizable to the US population with CKD and T2D, although strategies to address access and expand uptake are still needed. The pivotal phase 3 finerenone data and associated subanalyses of those data provide a rationale for the use of finerenone in a broad spectrum of patients and may prevent new-onset CV disease, including HF and AF. Results from the FINEARTS-HF phase 3 trial have recently been published [102], which showed a significantly lower rate of a composite of total worsening HF events and death from CV causes with finerenone vs. placebo in patients with HF and mildly reduced or preserved ejection fraction. In addition to further investigations of the effects of finerenone in novel combinations and in real-world clinical practice, we look forward to data becoming available in further patient populations. 

## Figures and Tables

**Table 1:** Overview of the key published finerenone phase 3 data for the treatment of CKD associated with T2D.

Trial ID	Population	Treatment Arms	Median	Primary Endpoint	Key Secondary Endpoints	**AEs of Special Interest**
			Follow-Up	(Finerenone vs. Placebo)	(Finerenone vs. Placebo)	**(Finerenone vs. Placebo)**
FIDELIO-DKD analyses						
FIDELIO-DKD primary analysis [18]	T2D and CKD treated with an ACEi/ARB (max dose without unacceptable side effects)	Finerenone + ACEi/ARB (N = 2833) vs. placebo + ACEi/ARB (N = 2841)	2.6 y	Time to kidney failure, a sustained decrease of ≥40% in eGFR from baseline over a period of ≥4 weeks, or death from renal causes 17.8% vs. 21.1% (HR 0.82 [95% CI: 0.73–0.93]; *p* = 0.001).	Time to death from CV causes, nonfatal MI, nonfatal stroke, or HHF 13.0% vs. 14.8% (HR 0.86 [95% CI: 0.75–0.99]; *p* = 0.03).	Hyperkalemia (related to study drug): 11.8% vs. 4.8% Serious hyperkalemia: 1.6% vs. 0.4% Permanent discontinuation because of hyperkalemia: 2.3% vs. 0.9% No sexual side effect reported
Prespecified subgroup analysis by history of HF [65]	History of HF With (n = 436) Without (n = 5238)	Finerenone + ACEi/ARB vs. placebo + ACEi/ARB	2.6 y	History of HF Time to kidney failure, a sustained decrease of ≥40% in eGFR from baseline over a period of ≥4 weeks, or death from renal causes With: 19.0% vs. 22.8% (HR 0.79 [95% CI: 0.52–1.20]); Without: 17.7% vs. 21.0%. (HR 0.83 [95% CI: 0.73–0.94])	History of HF Time to death from CV causes, nonfatal MI, nonfatal stroke, or HHF With: 23.6% vs. 29.5% (HR 0.72 [95% CI: 0.49–1.05]); Without: 12.2% vs. 13.4% (HR 0.90 [95% CI: 0.77–1.04]).	
Subgroup analysis by office SBP [67]	Baseline office SBP quartiles, mmHg Q1 (≤128.7; n = 1448) Q2 (>128.7 to ≤138.3; n = 1346) Q3 (>138.3 to ≤148.0; n = 1492) Q4 (>148.0; n = 1383)	Finerenone + ACEi/ARB vs. placebo + ACEi/ARB	2.6 y	Baseline office SBP quartiles Time to kidney failure, a sustained decrease of ≥40% in eGFR from baseline over a period of ≥4 weeks, or death from renal causes Q1: 12.9% vs. 14.7% (HR 0.87 [95% CI: 0.66–1.15]); Q2: 16.2% vs. 20.9% (HR 0.76 [95% CI: 0.59–0.98]); Q3: 18.4% vs. 22.0% (HR 0.81 [95% CI: 0.65–1.02]); Q4: 23.9% vs. 27.0% (HR 0.86 [95% CI: 0.70–1.07]).	Baseline office SBP quartiles Time to death from CV causes, nonfatal MI, nonfatal stroke, or HHF Q1: 10.9% vs. 11.4% (HR 0.95 [95% CI, 0.69–1.29]); Q2: 11.9% vs. 14.2% (HR 0.81 [95% CI: 0.60–1.10]); Q3: 13.2% vs. 16.1% (HR 0.79 [95% CI: 0.60–1.03]); Q4: 15.7% vs. 17.4% (HR 0.91 [95% CI: 0.70–1.18]).	
Subgroup analysis by history of AFF [66]	History of AFF With (n = 461) Without (n = 5213)	Finerenone + ACEi/ARB vs. placebo + ACEi/ARB	2.6 y	History of AFF Time to kidney failure, a sustained decrease of ≥40% in eGFR from baseline over a period of ≥4 weeks, or death from renal causes With: 15.8% vs. 15.8% (HR 1.13 [95% CI: 0.71–1.79]); Without: 18.0% vs. 21.6% (HR 0.81 [95% CI: 0.71–0.91]).	History of AFF Time to death from CV causes, nonfatal MI, nonfatal stroke, or HHF With: 26.7% vs. 29.9% (HR 0.88 [95% CI: 0.73–0.99]); Without: 11.7% vs. 13.5% (HR 0.85 [95% CI: 0.73–0.99]).	
Subgroup analysis by baseline HbA_1c_ and insulin use [64]	Baseline HbA_1c_ <7.5% (n = 2794) ≥7.5% (v = 2869) Baseline insulin use Yes (n = 2037) No (n = 3637)	Finerenone + ACEi/ARB vs. placebo + ACEi/ARB	2.6 y	Baseline HbA_1c_ Time to kidney failure, a sustained decrease of ≥40% in eGFR from baseline over a period of ≥4 weeks, or death from renal causes <7.5%: 18.8% vs. 21.6% (HR 0.86 [95% CI: 0.73–1.02]); ≥7.5%: 16.9% vs. 20.7% (HR 0.78 [95% CI: 0.66–0.93]). Baseline insulin use Time to kidney failure, a sustained decrease of ≥40% in eGFR from baseline over a period of ≥4 weeks, or death from renal causes Yes: 18.0% vs. 21.3% (HR 0.85 [95% CI: 0.73–0.98]); No: 17.4% vs. 20.8% (HR 0.79 [95% CI: 0.64–0.96]).	Baseline HbA_1c_ Time to death from CV causes, nonfatal MI, nonfatal stroke, or HHF <7.5%: 11.8% vs. 13.3% (HR 0.88 [95% CI: 0.71–1.09]); ≥7.5%: 13.9% vs. 16.3% (HR 0.83 [95% CI: 0.69–1.01]). Baseline insulin use Time to death from CV causes, nonfatal MI, nonfatal stroke, or HHF Yes: 13.8% vs. 16.7% (HR 0.82 [95% CI: 0.69–0.97]); No: 11.3% vs. 11.6% (HR 0.95 [95% CI: 0.74–1.23]).	
FIGARO-DKD analyses						
FIGARO-DKD primary analysis [19]	T2D and CKD treated with an ACEi/ARB (max dose without unacceptable side effects)	Finerenone + ACEi/ARB (N = 3686) vs. placebo + ACEi/ARB (N = 3666)	3.4 y	Time to death from CV causes, nonfatal MI, nonfatal stroke, or HHF 12.4% vs. 14.2% (HR 0.87 [95% CI: 0.76–0.98]; *p* = 0.03).	Time to kidney failure, a sustained decrease of ≥40% in eGFR from baseline over a period of ≥4 weeks, or death from renal causes 9.5% vs. 10.8% (HR 0.87 [95% CI: 0.76–1.01]).	Hyperkalemia (related to study drug): 6.5% vs. 3.1% Serious hyperkalemia: 0.7% vs. 0.1% No sexual side effect reported
Prespecified analysis of HF outcomes [68]	T2D and CKD treated with an ACEi/ARB (max dose without unacceptable side effects), without a history of symptomatic HFrEF	Finerenone + ACEi/ARB vs. placebo + ACEi/ARB	3.4 y	–	Risk for new-onset HF HR 0.68 [95% CI: 0.50–0.93]; *p* = 0.016). Risk for CV death or first HHF HR 0.82 [95% CI: 0.70–0.95]; *p* = 0.011). Risk for HF-related death or first HHF HR 0.68 [95% CI: 0.54–0.86]; *p* = 0.001). Risk for first HHF HR 0.71 [95% CI: 0.56–0.90]; *p* = 0.004). Risk for CV death or total (first or recurrent) HHF RR 0.79 [95% CI: 0.66–0.95]; *p* = 0.013). Risk for HF-related death or total HHF RR 0.70 [95% CI: 0.53–0.93]; *p* = 0.01). Risk for total hospitalization for HF RR 0.70 [95% CI: 0.52–0.94]; *p* = 0.018).	–
Exploratory, prespecified UACR subgroup analysis of kidney and CV composite endpoints [69]	UACR subgroups, mg/g 30 to <300 (n = 3414) ≥300 (v = 3729)	Finerenone + ACEi/ARB vs. placebo + ACEi/ARB	3.4 y	UACR subgroups, mg/g Time to death from CV causes, nonfatal MI, nonfatal stroke, or HHF 30 to <300: 13.1% vs. 14.9% (HR 0.87 [95% CI: 0.73–1.04]); ≥300: 12.0% vs. 13.5% (HR 0.90 [95% CI: 0.75–1.08]).	UACR subgroups, mg/g Time to kidney failure, a sustained decrease of ≥40% in eGFR from baseline over a period of ≥4 weeks, or death from renal causes 30 to 300: 8.4% vs. 7.3% (HR 1.16 [95% CI: 0.91–1.47]); ≥300: 10.9% vs. 14.3% (HR 0.74 [95% CI: 0.62–0.90]). Time to kidney failure, a sustained decrease of ≥57% in eGFR from baseline over a period of ≥4 weeks, or death from renal causes 30 to 300: 2.0% vs. 1.9% (HR 1.05 [95% CI: 0.65–1.71]); ≥300: 3.9% vs. 5.6% (HR 0.69 [95% CI: 0.51–0.93]).	
FIDELITY analyses						
FIDELITY [63]	T2D and CKD treated with an ACEi/ARB (max dose without unacceptable side effects)	Finerenone + ACEi/ARB (N = 6519) vs. placebo + ACEi/ARB (N = 6507)	3.0 y	Time to kidney failure, a sustained decrease of ≥57% in eGFR from baseline over a period of ≥4 weeks, or death from renal causes 5.5% vs. 7.1% (HR 0.77 [95% CI: 0.67–0.88]; *p* < 0.001). Time to death from CV causes, nonfatal MI, nonfatal stroke, or HHF 12.7% vs. 14.4% (HR 0.86 [95% CI: 0.78–0.95]; *p* = 0.0018).	Time to kidney failure, a sustained decrease of ≥40% in eGFR from baseline over a period of ≥4 weeks, or death from renal causes 13.1% vs. 15.3% (HR 0.85 [95% CI: 0.77–0.93]; *p* = 0.0004). Time to all-cause mortality 8.5% vs. 9.4% (HR 0.89 [95% CI: 0.79 to >1.00]). Time to all-cause hospitalization 43.5% vs. 45.0% (HR 0.96 [95% CI: 0.91–1.01]).	Hyperkalemia (related to study drug): 8.8% vs. 3.8% Serious hyperkalemia: 1.1% vs. 0.2% Permanent discontinuation because of hyperkalemia: 1.7% vs. 0.6% No sexual side effect reported
Prespecified on-treatment analysis * [70]	T2D and CKD treated with an ACEi/ARB (max dose without unacceptable side effects)	Finerenone + ACEi/ARB vs. placebo + ACEi/ARB	3.0 y	–	Risk for all-cause mortality 4.3% vs. 5.3% (HR 0.82 [95% CI: 0.70–0.96]; *p* = 0.014). Risk for CV mortality 2.9% vs. 3.6% (HR 0.82 [95% CI: 0.67–0.99]; *p* = 0.04). Renal death <0.1% vs. <0.1% (HR 0.53 [95% CI: 0.10–2.91]). Non-CV/nonrenal death 3.5% vs. 3.8% (HR 0.92 [95% CI: 0.77–1.10]).	–
Exploratory subgroup analysis of HF outcomes by eGFR/UACR [71]	UACR subgroups, mg/g < 300 (n = 4329) ≥300 (v = 8692) eGFR subgroups, mL/min/1.73 m^2^ ≥60 (v = 5195) < 60 (n = 7828)	Finerenone + ACEi/ARB vs. placebo + ACEi/ARB	3.0 y	–	Overall	
					Time to first HHF 3.9% vs. 5.0% (HR 0.78 [95% CI: 0.66–0.92]). Time to CV death or first HHF 8.3% vs. 9.8% (HR 0.83 [95% CI: 0.74–0.93]). UACR subgroups, mg/g Time to first HHF <300: 3.1% vs. 4.4% (HR 0.71 [95% CI: 0.52–0.97]); ≥300: 4.3% vs. 5.3% (HR 0.83 [95% CI: 0.68–1.00]). Time to CV death or first HHF <300: 7.9% vs. 9.7% (HR 0.80 [95% CI: 0.66–0.99]); ≥300: 8.4% vs. 9.9% (HR 0.86 [95% CI: 0.75–0.99]). eGFR subgroups, mL/min/1.73 m^2^ Time to first HHF ≥60: 2.9% vs. 4.2% (HR 0.69 [95% CI: 0.52–0.93]); <60: 4.6% vs. 5.5% (HR 0.84 [95% CI: 0.69–1.02]). Time to CV death or first HHF ≥60: 6.9% vs. 9.1% (HR 0.74 [95% CI: 0.61–0.90]); <60: 9.2% vs. 10.3% (HR 0.89 [95% CI: 0.77–1.02]).	
Prespecified subgroup analysis by history of ASCVD [72]	History of ASCVD With (n = 5935) Without (n = 7091)	Finerenone + ACEi/ARB vs. placebo + ACEi/ARB	3.0 y	History of ASCVD Time to death from CV causes, nonfatal MI, nonfatal stroke, or HHF With: HR 0.83 [95% CI: 0.74–0.94]; Without: HR 0.91 [95% CI: 0.78–1.06]. Time to kidney failure, a sustained decrease of ≥57% in eGFR from baseline over a period of ≥4 weeks, or death from renal causes With: HR 0.71 [95% CI: 0.57–0.88]; Without: HR 0.81 [95% CI: 0.68–0.97].	History of ASCVD Time to CV death or first HHF With: HR 0.82 [95% CI: 0.71–0.94]; Without: HR 0.86 [95% CI: 0.71–1.04]. Time to death from any cause With: HR 0.85 [95% CI: 0.74–0.99]; Without: HR 0.95 [95% CI: 0.79–1.14].	

* Events that occurred while on treatment and for ≥30 days after the last intake of study medication. ACEi: angiotensin-converting enzyme inhibitor; AE: adverse event; AFF: atrial fibrillation/flutter; ARB: angiotensin receptor blocker; ASCVD: atherosclerotic cardiovascular disease; CKD: chronic kidney disease; CV: cardiovascular; eGFR: estimated glomerular filtration rate; HbA1c: glycated hemoglobin; HF: heart failure; HFrEF: heart failure with reduced ejection fraction; HHF: hospitalization for heart failure; HR: hazard ratio; MI: myocardial infarction; Q: quartile; RR: rate ratio; SBP: systolic blood pressure; T2D: type 2 diabetes; UACR: urinary albumin-to-creatine ratio.

**Table 2:** Overview of ongoing randomized, controlled trials beyond the FIDELIO-DKD and FIGARO-DKD clinical trials of finerenone in CKD associated with T2D and Heart Failure *.

Trial ID	Phase	Population (Estimated/Actual Enrollment)	Treatment Arms	Primary Endpoint	Study Start Date	Estimated Study Completion
CVD trials						
REDEFINE-HF [NCT06008197]	3	Patients hospitalized with acute decompensated HF and mildly reduced or preserved LVEF (N = ~5200)	Finerenone vs. placebo	Total (first and subsequent) HHF, urgent visits for worsening HF, and CV deaths AEs leading to study drug discontinuation	November 2023	April 2026
CONFIRMATION-HF [NCT06024746]	3	Patients hospitalized for HF (N = ~1500)	Finerenone + empagliflozin vs. SOC (placebo)	Clinical benefit ^†^ SAEs or AEs leading to study drug discontinuation	February 2024	August 2025
FINALITY-HF [NCT06033950]	3	Patients with HFrEF who are intolerant or ineligible to receive treatment with sMRA (N = ~2600)	Finerenone vs. placebo	Time to first CV death or HF event SAEs or AEs leading to study drug discontinuation	June 2024	November 2027
FINEARTS-HF [NCT04435626]	3	Patients with HF and LVEF ≥40% (N = 6016)	Finerenone vs. placebo	CV deaths and HF events (first and recurrent)	September 2020	July 2024 (completed)
Kidney disease trials						
FIND-CKD [NCT05047263]	3	Nondiabetic CKD (N = 1574)	Finerenone vs. placebo	Change in eGFR	September 2021	February 2026
FINE-ONE [NCT05901831]	3	CKD associated with T1D (N = ~220)	Finerenone vs. placebo	Change in UACR	March 2024	October 2025
FIONA [NCT05196035]	3	Children with CKD (and proteinuria) (N = ~219)	Finerenone + ACEi/ARB vs. placebo + ACEi/ARB	Proportion with UPCR reduction of ≥ 30%	March 2022	March 2027
FIONA OLE [NCT05457283]	3 (OLE)	Children with CKD (and proteinuria) who completed the FIONA study (N = ~100)	Finerenone + ACEi/ARB	TEAEs, change in serum K+ levels, change in SBP	November 2022	September 2028
CONFIDENCE [NCT05254002]	2	CKD associated with T2D (N = ~807)	Finerenone + placebo Empagliflozin + placebo Finerenone + empagliflozin + placebo (combination vs. single drug)	(1) Relative change from baseline in UACR at 180 days in combination therapy group vs. empagliflozin alone, and (2) relative change from baseline in UACR at 180 days in combination therapy group vs. finerenone alone	June 2022	February 2025 (completed)
EFFEKTOR [NCT06059664]	2	Kidney transplant recipients (N = ~150)	Finerenone vs. placebo	Total number of participants who were eligible and enrolled in the main clinical trial and/or kidney biopsy substudy	November 2023	December 2025
FIVE-STAR [NCT05887817]	4	CKD associated with T2D (N = ~100)	Finerenone vs. placebo	Change in CAVI at 24 weeks after initiation of placebo or finerenone vs. baseline (CAVI is a physiological marker of arterial function)	September 2023	July 2026
Observational studies ^‡^						
FLAMINGO [NCT05640180]	Observational	CKD associated with T2D (N = ~3000)	Effects of treatment combination of finerenone + SGLT2is in routine medical care	(1) Time to first occurrence of the composite of onset of kidney failure, a sustained decrease of eGFR ≥40% from baseline over at least 4 weeks, or renal death; and (2) time to first occurrence of the composite endpoint of CV death or nonfatal CV event	November 2022	December 2023 (completed)
FINE-REAL [NCT05348733]	Observational	Initiated treatment with finerenone for T2D and CKD (N = ~5500)	Finerenone	Description of clinical characteristics and treatment patterns	June 2022	January 2028
FIRST-2 [NCT05703880]	Observational	Initiated treatment with finerenone for T2D and CKD (N = ~10,000)	Finerenone	Description of clinical characteristics and treatment patterns	June 2023	July 2024 (completed)
FINEGUST [NCT05526157]	Observational	Receiving or initiating treatment for CKD or T2D (N = ~50,000)	Finerenone, SGLT2is, GLP-1 RAs, sMRAs, ns-MRAs	Description of clinical characteristics and treatment patterns	October 2022	September 2024 (completed)
RW study in HFrEF [NCT05974566]	Observational	HFrEF (N = ~60)	Finerenone	Change in serum NT-proBNP levels	August 2023	October 2023 (not yet recruiting)
IN-REALITY [NCT06763146]	Observational	CKD and T2D (N = not provided)	Finerenone	Safety and tolerability of finerenone	May 2025	October 2025
FIRST 2.5 [NCT06608212]	Observational	CKD and T2D Finerenone use in routine medical care (US) N = ~150,000	Finerenone	Time to composite CV outcome	October 2024	June 2025

Further details for all studies included in the table can be found at https://clinicaltrials.gov/. * Clinical trials in aldosteronism or other indications excluded. ^†^ Clinical benefit defined as time to death from any cause, number of HF events, time to first HF event, difference of ≥5 points on the Kansas City Cardiomyopathy Questionnaire—Total Symptom Score (KCCQ-TSS). ^‡^ Excludes trials taking place exclusively in Japan or China. ACEi: angiotensin-converting enzyme inhibitor; AE: adverse event; ARB: angiotensin receptor blocker; CAVI: cardio-ankle vascular index; CKD: chronic kidney disease; CVD: cardiovascular disease; eGFR: estimated glomerular filtration rate; GLP-1 RA: glucagon-like peptide-1 receptor agonist; HF: heart failure; HFrEF: heart failure with reduced ejection fraction; HHF: hospitalization for heart failure; LVEF: left ventricular ejection fraction; ns-MRA: nonsteroidal mineralocorticoid receptor antagonist; OLE: open-label extension; RW: real-world; SAE: serious adverse event; SBP: systolic blood pressure; SGLT2i: sodium-glucose cotransporter-2 inhibitor; sMRA: steroidal mineralocorticoid receptor antagonist; SOC: standard of care; T1D: type 1 diabetes; T2D: type 2 diabetes; TEAE: treatment-emergent adverse event; UACR: urinary albumin-to-creatine ratio; UPCR: urinary protein-to-creatinine ratio.

## Supplementary Materials

The following supporting information can be downloaded at https://www.mdpi.com/article/10.3390/jcm14093213/s1. A plain language summary intended for use by non-specialist medical professionals and patients is included with this article in Supplementary Materials (S1).
