# Supplementary material for: Finerenone: Potential Clinical Application Across the Spectrum of Cardiovascular Disease and Chronic Kidney Disease
Source: J Clin Med. 2025 May 6;14(9):3213. doi: 10.3390/jcm14093213 (PMC12539649; doi:10.3390/jcm14093213)
Supplement: Supplementary file 1 [file jcm-14-03213-s001.zip › jcm-3491551-supplementary.pdf]

Supplementary Material

Plain language summary

People living with type 2 diabetes (T2D for short) are at risk of developing a complication of their diabetes called chronic kidney disease (CKD for short). Some people with CKD and T2D will also develop problems with their heart and blood vessels. CKD gets worse over time (called disease progression), with some people eventually needing a kidney transplant or to use a machine that does the job of their kidneys (called dialysis). Finerenone is a drug used to treat CKD in people who also have T2D. Finerenone slows how quickly a person's CKD gets worse and may also benefit their heart. An important cause of CKD is overactivity of a protein found inside many cells called the mineralocorticoid receptor. This overactivity of the mineralocorticoid receptor damages the kidneys and the heart so they do not work as well as they should. Finerenone stops the mineralocorticoid receptor from being overactive. This review article talks about how common T2D and CKD are and gives an overview of what finerenone is, where the results supporting its use in CKD and T2D come from, and what these results mean for people living with CKD and T2D. This review also looks at other types of diseases (such as type 1 diabetes or a serious heart condition called heart failure) where finerenone might be helpful and is currently being tested or has just finished being tested in clinical trials involving many people with the disease.
